# Evaluation of the factor structure of the Russian version of PID-5-BF

**DOI:** 10.1192/j.eurpsy.2022.948

**Published:** 2022-09-01

**Authors:** M. Zinchuk, G. Kustov, A. Gersamija, A. Yakovlev, E. Pashnin, N. Voinova, S. Popova, A. Guekht

**Affiliations:** 1 Moscow Research and Clinical Center for Neuropsychiatry, Suicide Research And Prevention, Moscow, Russian Federation; 2 Moscow Research and Clinical Center for Neuropsychiatry of the Healthcare Department of Moscow, Russian Federation, Department Of The Suicidal Researchs, Moscow, Russian Federation

**Keywords:** Psychometrics, alternative model of personality disorders (AMPD), Personality Inventory for DSM-5 Brief Form (PID-5-BF), dimensional model

## Abstract

**Introduction:**

Traditional categorical classifications of personality disorders (PD) have been criticized for insufficient structural and cross-cultural validity. In the DSM-5 Section III, alternative model of the PDs (AMPD), the maladaptive personality traits are divided into five domains: negative affect, detachment, antagonism, disinhibition and psychoticism. The Personality Inventory for DSM-5 Brief Form (PID-5-BF) is a 25-item self-report questionnaire that measures the severity of each of these five domains. To date, no questionnaires assessing pathological personality traits following the AMPD have been validated in Russia.

**Objectives:**

This study aimed to investigate the factor structure of the Russian version of PID-5-BF.

**Methods:**

Five hundred and 86 (female - 505 (86,2%), age - 18–77 years (M - 28.2, SD - 11.5)) consecutive inpatients with non-psychotic mental disorders were assessed with the Russian language version of the PID-5-BF. Exploratory structural equation modelling (ESEM) with Robust Diagonally Weighted Least Squares method of extraction and Robust Equamax rotation was performed in Factor v 11.04.02.

**Results:**

The results of the ESEM analysis showed good fit of the five-factor model (CFI - 0.982; TLI - 0.971; RMSEA (95% CI) - 0.036 (0.01-0.05). Most of the items had the highest factor loadings on their mother domains. However, two items showed weak loadings on their designated factors (<0.4), and attention seeking item had a primary load to (low) detachment instead of antagonism.

**Conclusions:**

The PID-5-BF was found to be a valid and reliable tool for the evaluation of the AMPD trait domain

**Disclosure:**

No significant relationships.

